# Lipodendriplexes mediated enhanced gene delivery: a cellular to pre-clinical investigation

**DOI:** 10.1038/s41598-020-78123-6

**Published:** 2020-12-08

**Authors:** Imran Tariq, Muhammad Yasir Ali, Muhammad Farhan Sohail, Muhammad Umair Amin, Sajid Ali, Nadeem Irfan Bukhari, Abida Raza, Shashank Reddy Pinnapireddy, Jens Schäfer, Udo Bakowsky

**Affiliations:** 1 Department of Pharmaceutics and Biopharmaceutics, University of Marburg, Robert-Koch-Str. 4, 35037 Marburg, Germany; 2 Punjab University College of Pharmacy, University of the Punjab, Allama Iqbal Campus, Lahore, 54000 Pakistan; 3 Department of Pharmaceutics, Faculty of Pharmaceutical Sciences, GC University Faisalabad, Faisalabad, Pakistan; 4 Riphah Institute of Pharmaceutical Sciences, Riphah International University, Lahore Campus, Lahore, Pakistan; 5 National Institute of Lasers and Optronics College, PIEAS, Islamabad, Pakistan; 6 CSL Behring GmbH, Emil-von-Behring-Str. 76, 35041 Marburg, Germany; 7 Department of Pharmacy, Faculty of Health and Medical Sciences, University of Copenhagen, Copenhagen, Denmark

**Keywords:** Biotechnology, Cancer, Drug discovery, Oncology, Nanoscience and technology, DNA nanotechnology, Nanobiotechnology, Nanomedicine, Nanoscale materials

## Abstract

Clinical success of effective gene therapy is mainly hampered by the insufficiency of safe and efficient internalization of a transgene to the targeted cellular site. Therefore, the development of a safe and efficient nanocarrier system is one of the fundamental challenges to transfer the therapeutic genes to the diseased cells. Polyamidoamine (PAMAM) dendrimer has been used as an efficient non-viral gene vector (dendriplexes) but the toxicity and unusual biodistribution induced by the terminal amino groups (–NH_2_) limit its in vivo applications. Hence, a state of the art lipid modification with PAMAM based gene carrier (lipodendriplexes) was planned to investigate theirs in vitro (2D and 3D cell culture) and in vivo behaviour. In vitro pDNA transfection, lactate dehydrogenase (LDH) release, reactive oxygen species (ROS) generation, cellular protein contents, live/dead staining and apoptosis were studied in 2D cell culture of HEK-293 cells while GFP transfection, 3D cell viability and live/dead staining of spheroids were performed in its 3D cell culture. Acute toxicity studies including organ to body index ratio, hematological parameters, serum biochemistry, histopathological profiles and in vivo transgene expression were assessed in female BALB/c mice. The results suggested that, in comparison to dendriplexes the lipodendriplexes exhibited significant improvement of pDNA transfection (p < 0.001) with lower LDH release (p < 0.01) and ROS generation (p < 0.05). A substantially higher cellular protein content (p < 0.01) and cell viability were also observed in 2D culture. A strong GFP expression with an improved cell viability profile (p < 0.05) was indicated in lipodendriplexes treated 3D spheroids. In vivo archives showed the superiority of lipid-modified nanocarrier system, depicted a significant increase in green fluorescent protein (GFP) expression in the lungs (p < 0.01), heart (p < 0.001), liver (p < 0.001) and kidneys (p < 0.001) with improved serum biochemistry and hematological profile as compared to unmodified dendriplexes. No tissue necrosis was evident in the animal groups treated with lipid-shielded molecules. Therefore, a non-covalent conjugation of lipids with PAMAM based carrier system could be considered as a promising approach for an efficient and biocompatible gene delivery system.

## Introduction

Gene therapy is a promising strategy to deliver a therapeutic gene to the cells for the treatment of various acquired and inherited diseases, especially in cancer. Effective gene therapy is mainly restrained by the insufficiency of safe and efficient gene delivery systems. Thus, the main objective of gene therapy is to develop an efficient and non-toxic carrier system for the effective delivery of genetic material to the targeted cellular site^1–3^.

Viral or non-viral vectors are two basic approaches employed for therapeutic gene delivery. Viral vectors are the most efficient gene carriers but the induction of strong immune response, high cost, safety concerns and limitations in the size of the inserted gene restricts their applications in the biological system and urges for the development of a safe and economic alternative^4,5^. Contrarily, plasmid-based non-viral vectors have advantages over the viral vectors in terms of safety, ease of construction for large therapeutic genes and cost-effectiveness. However, the larger size, rapid enzymatic degradation as well as the initiation of an immune response can limit their cellular uptake and subsequent poor in vivo gene expression^6,7^. Therefore, a protective shielding of the naked nucleic acid can be accomplished by a stable complex formation with a positively charged non-viral vector system^8^. These stable complexes can thus, efficiently cross the cell membrane and release the cargo for the desired therapeutic effects^9^.

Among the polycationic non-viral vector systems, polymeric nanoparticles are gaining more attention in gene delivery research^10^. Cationic polymers having the different stoichiometry of linear, branched or dendritic structure with the ability to make a stable complex with negatively charged nucleic acid by their primary, tertiary or quaternary amine groups. Therefore, the flexibility, monodispersity, reproducibility and facile manufacturing of such polymers have make them potential candidates for gene therapy^11^.

**Figure 1:**
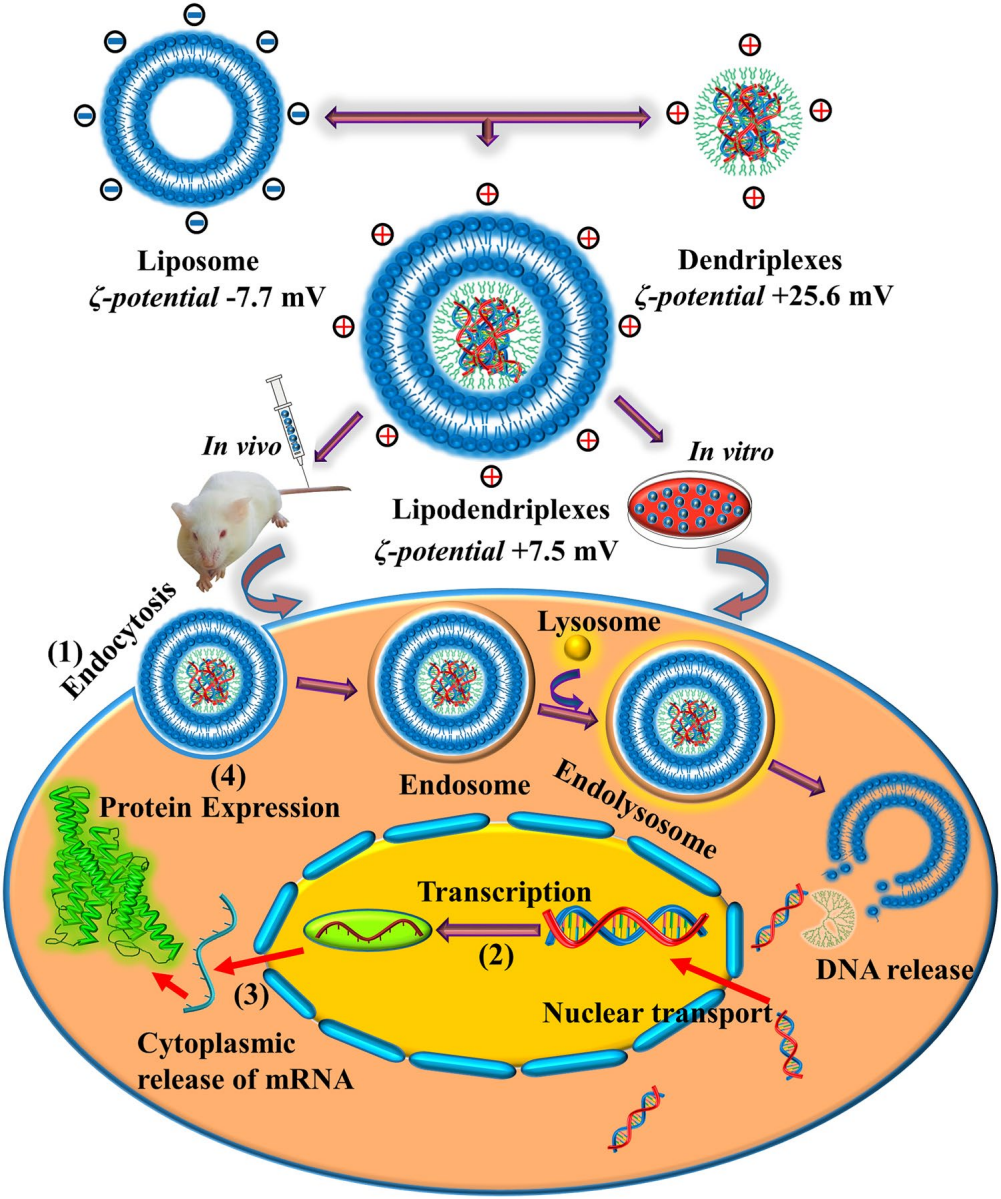
Schematic diagram of lipodendriplexes formation and its cellular internalization. (1) Endocytosis of lipodendriplexes and release of nucleic acid into the cytoplasm. (2) Transcription of gene-encoded DNA into mRNA. (3) Export of mRNA from the nucleus to the cytoplasm. (4) Protein expression.

Among the synthetic polymeric systems, polyamidoamine (PAMAM) dendrimer and polyethylenimine (PEI) are off-the-shelf polymers and have most extensively been used in recent years^12^. In comparison to PEI, the PAMAM dendrimer is a safe^13,14^ and well-characterized cationic system and has been used for efficient nucleic acid delivery^15,16^. The primary amine groups of the PAMAM dendrimer mostly participate in the interaction with the nucleic acid to make stable nano-complexes (dendriplexes), while the tertiary amino groups act as proton sponge in the endosomal environment to facilitate the release of cargo in the cytoplasm^17–19^. Nevertheless, polycationic nature of PAMAM dendrimer can contribute to cytotoxicity, which is one of the drawbacks associated with this polymeric system. Additionally, the interaction of PAMAM dendrimer with oppositely charged macromolecules in plasma (like heparin) may lead to the displacement of nucleic acid from the complexes which may result in lower gene expression^20^. Some researchers also reported the unusual biodistribution of higher generation PAMAMs in mice including short plasma circulation time, high hepatic or renal clearance, and toxicity in vital organs^21^. Many strategies have been proposed to mask the terminal amino groups inherited toxicity of PAMAM dendrimer by modifying their physicochemical properties and make them a more suitable candidate for systemic application^22–24^. Lipid-based gene delivery can be a safe alternative to viral vectors to deliver nucleic acid or proteins to target cells in preclinical models^25^. Therefore, a non-covalent hybridization of dendriplexes with the liposomal membrane (lipodendriplexes) is a useful tool to avoid the demerits associated with the naked dendriplexes system. The inner lipid layer of the resulting complex, not only confers the biocompatibility to the naked dendriplexes but also decreases the chances of degradation in an outer environment, while the outer lipid layer provides the steric stabilization and assists in the attachment to the cellular site to facilitate the release of cargo^26^ (Fig. 1). By doing so, they can exhibit negligible hemolytic toxicity and higher transfection efficiency with better in vivo tolerance, as compared to the naked dendrimeric system^27^.

According to the best of our knowledge, the current work is the first comprehensive study to explore the gene expression in 3D cell culture along the biodistribution and acute toxicity profile by a PAMAM based lipodendriplexes system. Optimized lipodendriplexes were prepared by the incorporation of liposomes (dipalmitoylphosphatidylcholine (DPPC) and cholesterol (CH) combination at the molar ratio of 85:15) with PAMAM based dendriplexes system. In vitro experiments were performed to study the physicochemical characteristics, transfection efficiency and cytotoxicity measurements both in 2D and 3D cell cultures. Afterward, a comprehensive acute toxicity profile and in vivo biodistribution of optimized complexes were studied using female BALB/c mice*.* The in vitro profile was then correlated with in vivo results to get the full benefit of optimized lipid triblock nanocarrier system.

## Materials and methods

1,2-Dipalmitoylphosphatidylcholine (DPPC) was gifted from Lipoid AG (Steinhausen, Switzerland). Cholesterol (CH), PAMAM dendrimer; ethylenediamine core, generation 5.0 solution (5 wt% in methanol), 4-(2-hydroxyethyl)-1-piperazineethanesulfonic acid (HEPES) and 2,7-dichlorofluorescin diacetate (H_2_DCFDA) were obtained from Sigma Aldrich Chemie GmbH (Taufkirchen, Germany). Pierce protein BCA assay kit and Gene JET Plasmid Miniprep kit were purchased from Thermo Fischer Scientific (Dreieich, Germany). Promokine Calcein-AM and PromoKine LDH cytotoxicity kit II was purchased from Promo Cell GmbH (Darmstadt, Germany). CellTiter-Glo 3D Reagent and Cell culture lysate reagent (CCLR) was purchased from Promega Corporation (Mannheim, Germany). Dulbecco's modified Eagle's minimum essential medium (DMEM), Fetal bovine serum (FBS) were purchased from Capricon (Ebsdorfergrund, Germany). Purified water from PURELAB flex II dispenser (ELGA Lab Waters, High Wycombe, UK) was used for different experiments. All other reagents used in experiments were of analytical grade.

### Plasmid DNA and their amplification

The pCMV-luc (ampicillin-resistant) encoding firefly luciferase (pDNA) and pCMV-GFP (kanamycin-resistant) with green fluorescent protein-expressing plasmids were obtained from Plasmid Factory (Bielefeld, Germany). Plasmids were amplified in *Escherichia* *coli* (DH5α strain) and purified using a Gene JET Plasmid Miniprep kit, according to the manufacturer’s protocol. The concentration and purity of nucleic acids were determined by A_260/280_ using Nano-100 (Allsheng, China). The integrity of plasmids was confirmed by 0.9% agarose gel electrophoresis and was stored at − 20 °C for further experiments.

### Cell culture

Human embryonic kidney (HEK-293) cells were obtained from American Type Culture Collection (ATCC, Manassas, USA) and cultured in DMEM (containing 10% of FBS and non-essential amino acids) at 37 °C under 5% CO_2_ humid conditions. All cells were grown to 80% confluency.

### Preparation of liposomes, dendriplexes, and lipodendriplexes

Liposomes were prepared by the thin-film hydration method, as described previously^28^. For the preparation of liposomal formulation, lipids of DPPC and cholesterol at the molar ratio of 85:15 were dissolved in chloroform:methanol (2:1, v/v) mixture. The organic phase was removed by a rotary evaporator (Heidolph, Germany) to get a thin lipid film. The lipid film was then hydrated with 20 mM HEPES-5% glucose buffer, pH 7.4 (HBG buffer) and sonicated (Elmasonic P30H, Elma Schmidbauer GmbH, Singen, Germany) for 10 min at 45 °C to get multilamellar liposomes. The liposomal suspension was then slowly extruded through a 100 nm polycarbonate membrane, using a pre-heated Avanti mini extruder (Avanti Polar Lipids, Alabaster, USA), to get a unilamellar liposomal suspension.

For dendriplexes, pDNA and PAMAM were dissolved in HBG buffer and were mixed by vigorous pipetting (equal volumes at the N/P ratio 12/1; the ratio of terminal amino groups in the PAMAM dendrimer to the phosphate groups of the nucleic acid) followed by incubation at room temperature for 30 min. Lipodendriplexes were formed by incubating dendriplexes with a fine liposomal dispersion in HBG buffer (liposome to PAMAM mass ratio 0.5/1) for 1 h (equal volumes).

## In vitro characterization and cell culture experiments

### Atomic force microscopy (AFM)

The surface morphology of the complexes was evaluated by atomic force microscopy using NanoWizard 3 NanoScience (JPK BioAFM, Bruker Nano GmbH, Berlin, Germany). A 20 µl diluted sample (1:100) of complexes was placed onto a silicon wafer and allowed to adsorb on the silica surface for 15 min. For the analysis, commercially available cantilever tips (NSC14 AlBS, Mikromasch Europe, Wetzlar, Germany) with a resonance frequency of about 160 kHz, having a length of 125 mm and nominal force constant of 5 N/m were used. The measurements were carried out using the intermittent contact mode (tapping mode) at a scan rate between 0.5 and 1.5 Hz. The surface roughness was evaluated as average root mean square (RMS) value Rq, using the height mode of AFM images. The results were analysed using JPK data processing software.

### Zeta potential (ζ) measurements

The zeta potential (ζ) of the complexes was assessed with laser Doppler velocimetry (LDV) by measuring the electrophoretic mobility at a scattering angle of 17° using Zetasizer Nano ZS (Malvern Instruments, Malvern, UK). The samples were diluted with purified water in a ratio of 1:100 and were analysed using a clear disposable folded capillary cell (DTS1060). Depending upon the intensity signals of the sample, the instrument automatically performs 15–100 runs per measurement. The average value was calculated with data of three individual formulations (mean ± standard deviation).

### pDNA transfection efficiency

For in vitro pDNA transfection experiments, HEK-293 cells were seeded at a density of 10,000 cells per well in 96-well microtiter plates and were incubated overnight. The cells were then incubated with the complexes containing 0.25 µg pDNA for 4 h, using DMEM (containing 10% of FBS and non-essential amino acids). Afterward, an additional medium was added to the wells and plates were further incubated for further 44 h. After the incubation period, the complexes were removed and cells were washed twice with phosphate buffer (PBS), pH 7.4 supplemented with Ca^2+^ and Mg^2+^. The buffer was then replaced with CCLR to ensure the cell lysis. The luminescence was detected by the reaction of cell lysate (containing the luciferase gene) with luciferase assay reagents (Synchem OHG, Felsberg, Germany).

Pierce protein BCA assay kit was used for protein quantification using bovine serum albumin (BSA) standard curve, according to the manufacturer’s protocol. A portion of lysate was incubated with BCA mixture at 37 °C for 30 min and the absorbance of resulting complexes was measured at 570 nm (FLUOStar Optima). The results obtained from these two experiments were expressed as relative luminescence unit (RLU) per mg protein.

### Lactate dehydrogenase (LDH) release assay

For LDH release assay, PromoKine LDH cytotoxicity kit II was used, according to the manufacturer’s instructions with little modifications. Briefly, HEK-293 cells were seeded at a density of 20,000 cells per well in 96-well microtiter plates. The cells were incubated with the complexes, containing 0.25 µg pDNA in each well, for 4 h using 1% Triton-X 100 as a high control. The cells without any treatment were considered as low control while the wells with the growth medium only were used as background control. The additional medium was added after 4 h and incubated for further 20 h. Afterward, 10 µl of supernatant was transferred to another clear 96-well microtiter plate and mixed with 100 µl LDH reaction mixture. The plate was incubated for 30 min at room temperature and the absorbance was then measured at 485 nm (FLUOStar Optima)^29,30^. The % cytotoxicity was determined using the following formula:

1 $$\mathrm{Cytotoxicity }\left(\mathrm{\%}\right)= \frac{\mathrm{Sample}-\mathrm{Low Control}}{\mathrm{High Control}-\mathrm{Low Control }} \times 100$$

### Determination of reactive oxygen species (ROS) generation

For ROS generation assay, HEK-293 cells were seeded at the seeding density of 25,000 cells per well in 96-well microtiter plates (black plate with clear bottom) and were incubated overnight before the experiment. For the measurement of ROS production, cellular ROS protocol by Abcam (Cambridge, UK) with slight modifications, was used. Briefly, 100 µl of 25 µM cell-permeant reagent 2,7-dichlorofluorescein diacetate (H_2_DCFDA) was added to the cells, 1 h before adding the complexes. After washing with PBS, the complexes containing 0.25 µg pDNA were added in each well and incubated for 1 h using 50 μM *tert*-butyl hydroperoxide (tBHP) as a positive control. The cells were washed again with PBS and detectable green fluorescence of dichlorofluorescein (DCF) was recorded at excitation and emission wavelengths of 485 nm and 520 nm, respectively (FLUOStar Optima). The intracellular fluorescence visualization was analysed using a microscope (CKX-53 Olympus, USA), equipped with GFP fluorescence detection filters (ex.505 nm–em.530 nm)^31–33^.

### Determination of cellular protein content

For cellular protein content determination, the complexes were incubated with the cells as mentioned in Sect. 3.3. The cell lysate was incubated with BCA mixture at 37 °C for 30 min and the absorbance of resulting complexes was measured at 570 nm. Results were expressed as a percentage of the control (untreated) cells^34^.

### Live/dead assay in 2D culture

For live/dead 2D cell viability assay, HEK-293 cells were seeded at the seeding density of 50,000 cells per well in 24-well plates and were incubated overnight before the experiment. The cells were incubated with the complexes containing 1 µg pDNA for 24 h. Afterward, cells were washed with PBS followed by the addition of Calcein-AM (2 μM for live cells) and propidium iodide (4 μM for dead cells). The cells were further incubated for 20 min at 37 °C and rewashed with PBS to remove the excess stain. The cells were then visualized under a fluorescence microscope equipped with green (ex.505 nm–em.530 nm) and red fluorescence filters (ex.550 nm–em.650 nm), respectively.

### Annexin/PI flow cytometry

For Annexin/PI flow cytometry analysis, 90,000 HEK-293 cells per well were seeded in 12-well cell culture plates. After overnight incubation, the cells were incubated with the complexes containing 2 μg of pDNA in each well for 4 h. Afterward, an additional medium was added and plates were further incubated for 44 h. The cells were then collected, washed with cold PBS and resuspended in 1 × binding buffer. A 50 μl of binding buffer supplemented with 5 μl (10 μg/ml) of FITC Annexin V were gently vortexed with an equal volume of cell suspension and incubated at room temperature for 15 min under dark. Then, 400 μl of binding buffer, containing 1 μl (2 mg/ml) of propidium iodide (PI) was added and placed on the ice for 5 min, before analysis by flow cytometer (Guava easyCyte, Millipore Sigma, USA). The data was processed by FlowJo 10 software.

### Preparation of 3D spheroids

For the preparation of spheroids, the cell suspension was grown on agarose coated plates. Briefly, a pre-heated, sterile 1% agarose solution was added into each well of a flat bottom 24 well plate and allowed to cool to form a concave surface. Afterward, a cell suspension of 5000 cells was transferred to each well of agarose coated plates. The plates were then sealed with sterile sealing tape and placed on an orbital shaker (KS4000 IC, IKA Werke, Staufen, Germany) to agitate the cells for 3 h, using a slow rotation speed of 40 rpm, at 37 °C. The cells were then allowed to incubate and monitored daily for the formation of spheroids. When the size of the spheroid reached a diameter of around 800–1000 μm, they were subjected to treatment with different complexes.

### GFP transfection in spheroids

For the GFP transfection assay, spheroids of HEK-293 cells were prepared by agarose coating technique as mentioned above. The spheroids were incubated with complexes containing 1 µg pDNA for 4 h. Afterward, an additional medium was added to the wells and plates were incubated for further 44 h. The GFP expression in spheroids was then visualized by a fluorescence microscope, equipped with GFP fluorescence detection filters.

### 3D cell viability assay

For 3D cell viability assay, the spheroids were incubated with the complexes containing 1 µg pDNA for 24 h. The assay was then performed using CellTiter-Glo 3D reagent according to the manufacturer protocol. Briefly, 50 μl of reagent was mixed with an equal volume of medium containing spheroid in a white opaque 96-well microplate and the suspension was shaken for 5 min using an orbital shaker to ensure the cell lysis. The cell lysate was then incubated at room temperature for 30 min (under dark condition). The luminescence signals of the samples were then recorded with the microplate reader (FLUOStar Optima)^35^.

### Live/dead assay 3D cell culture

To check the live/dead cell viability ratio in the spheroids, live/dead staining assay was performed using Calcein-AM (2 μM) and PI (4 μM) solutions. The spheroids were incubated with the complexes containing 1 µg pDNA for 24 h. After that, the spheroids were washed with PBS followed by incubation with live/dead staining solutions (in PBS) for 20 min at 37 °C with 5% CO_2_. The spheroids were then re-washed again with PBS to remove the excess stain and were visualized under a confocal laser scanning microscope (CLSM) using green and red fluorescence filters, respectively.

## In vivo experiments

### Experimental study design

**Figure 2:**
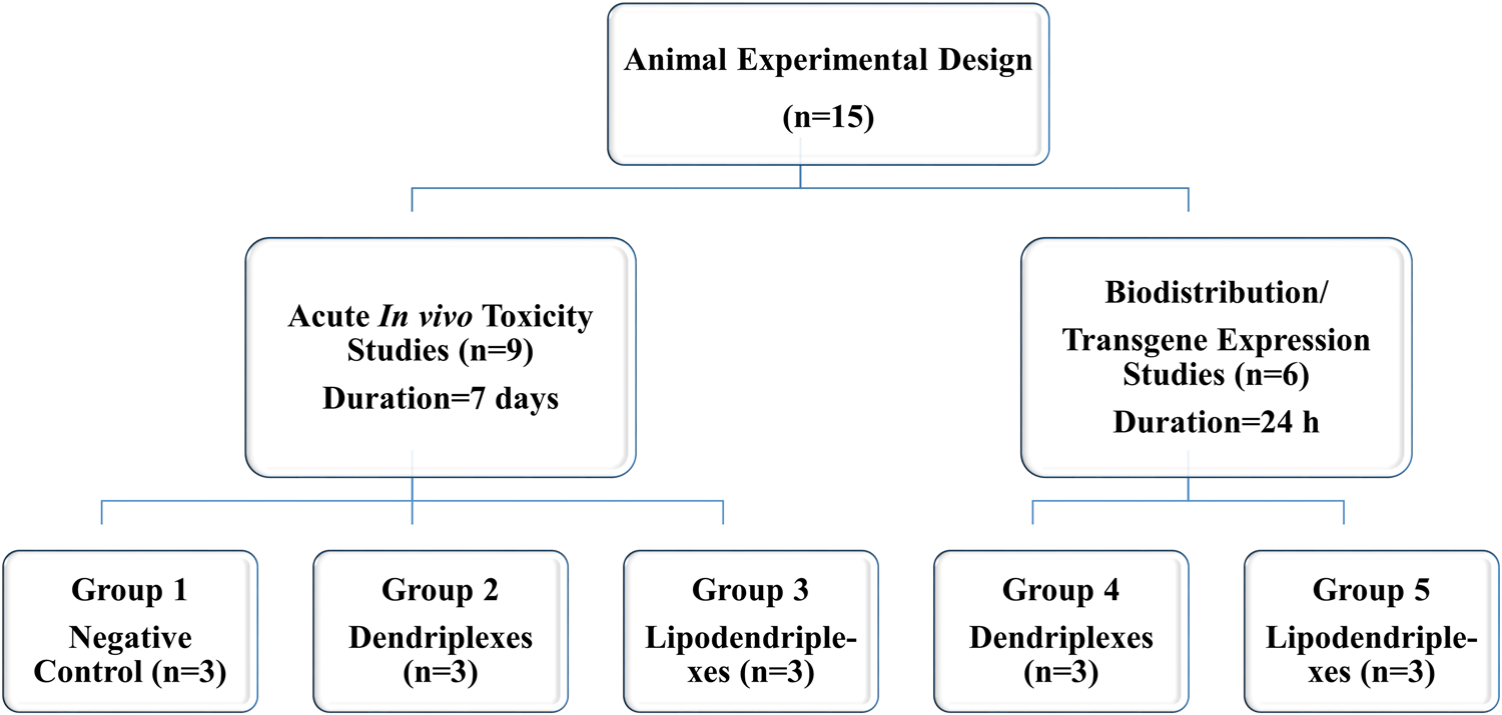
Animal experimental design: Schematic representation of acute in vivo toxicity (group 1–3) and biodistribution studies (group 4–5).

The 7–8 weeks old, female BALB/c mice were purchased from the National Institute of Health, Islamabad, Pakistan and handled according to the protocols approved by the Ethical committee of Punjab University College of Pharmacy, University of the Punjab (Protocol No. AEC/PUCP/1085 dated 03.09.2018). All the experimental work was in accordance with the institution’s ethical and Organization for Economic Cooperation and Development (OECD) 425 guidelines, except that *i.v.* administration was used. Total 15 animals were used in the study and were divided into 5 groups (each having 3 animals) according to the experimental design shown in Fig. 2. The animals were maintained in a controlled environment (between 20 and 25 °C; humidity 60 ± 10%; 12 h light/dark cycle) with the standard conditions of food and water (ad libitum).

### Acute in vivo toxicity

For in vivo acute toxicity studies, the untreated mice were served as a negative control (group 1), while the groups injected with the dendriplexes and lipodendriplexes (containing 10 µg pDNA) by tail vein were categorized into group 2 and group 3, respectively. The first dose of the complexes was administered on day 1 while the second dose was given on day 4. The mice were kept under observation for 7 days to monitor any change in weight, behaviour and physical alteration to assess any kind of illness. The following parameters were evaluated for acute in vivo toxicity determination:

#### Organ to body index ratio

The relative organ to body ratio was considered as a tool to evaluate any toxic effect of the complexes on mice organs after their repeated administration. The animals were euthanized by cervical dislocation method at day 7 and vital organs (i.e. liver, lungs, heart, kidney and spleen) were removed carefully. The organs were washed with normal saline and weighed individually for the organ to body index calculation, which is the ratio of the organ weight to the total body weight ratios^36^.

#### Serum biochemical and haematological parameters

To check the biocompatibility of the complexes, the blood was collected under anesthesia by the cardiac puncture at day 7 and the serum biochemical and hematological parameters were analysed. The serum was separated by centrifugation of the blood at 1200×*g* for 10 min (Centurion Scientific, Chichester, UK). The supernatant was collected and stored at − 20 °C for further experiments. Serum biochemical parameters like liver function tests (LFTs), renal function tests (RFTs), total bilirubin, blood glucose and total protein levels were evaluated using a serum biochemical marker analyser (Micro lab 300, Merck, Germany).

Hematology parameters, included the red blood cells count (RBCs), hemoglobin (Hb), hematocrit (HCT), white blood cells count (WBCs), mean corpuscular volume (MCV), neutrophils, eosinophils, lymphocytes, monocytes, platelets count (PLT), mean corpuscular hemoglobin (MCH), mean corpuscular hemoglobin concentration (MCHC), and mean platelet volume (MPV) were measured using a hematology analyser (Icon-3, Norma Instruments, Budapest, Hungary).

#### Erythrocytes aggregation assay

For the erythrocyte aggregation assay, erythrocytes were washed three times with PBS by centrifugation at 5000×*g* for 5 min. The erythrocyte pellet was then resuspended with PBS to make a final stock suspension of 2% (v/v). A 100 μl of the erythrocyte suspension was mixed with 100 μl of the complexes containing 1 μg pDNA and incubated for 2 h at room temperature. A 10 μl sample of the mixture was placed on a glass slide and observed under bright-field microscopy.

#### Histopathology of vital organs

The macroscopic necropsies were evaluated in the organ tissues of sacrificed mice. The vital organs like heart, lungs, liver and kidneys were collected for tissue histopathology and were fixed in 10% formalin solution before embedding into a paraffin block. Thin tissue sections (0.5 μm) were then obtained with the help of rotary microtome (Hunan Kaida scientific instruments, Changsha, China) and were stained using hematoxylin and eosin (H & E staining) dyes for microscopic evaluation (Olympus BX51M, Tokyo, Japan).

### In vivo biodistribution and ex vivo imaging experiments

For the in vivo biodistribution analysis, the mice were i.v. injected with the complexes (dendriplexes; group 4 and lipodendriplexes; group 5) containing pCMV-GFP (10 μg) by tail vein. The mice were sacrificed 24 h post injection and the vital organs were collected for ex vivo imaging. A UVP iBox Explorer2 (Analytik Jena US LLC, Jena, Germany) small animal imaging system, having customized wavelength filters for green fluorescence (ex.455 nm–495 nm, em.503 nm–523 nm) was used for the measurement of the GFP expression in vital organs. The results were analysed by using VisionWorks LS software.

### Frozen tissues GFP distribution analysis

The organs were stored in 10% formalin solution for the fixation of the tissues and were frozen at − 20 °C. GFP expression was then observed in the thin sections of frozen tissues under the fluorescence microscope (EVOS FL Cell Imaging System, Thermo Scientific, San Diego, CA, USA)*.*

### Statistical analysis

All the experimental evaluations were performed in triplicates and the values were expressed as mean ± standard deviation (SD). One-way ANOVA was performed to identify statistically significant differences using IBM SPSS software (Ver. 22). Dunnett’s test was used for multi-comparison between the results and control, whereas multi-comparison among the different groups was made using Tukey’s test. Probability values less than 0.05 were considered significant. Statistical significances are denoted as *p < 0.05, **p < 0.01, ***p < 0.001.

## Results and discussion

### In vitro characterization of dendriplexes and lipodendriplexes

**Figure 3:**
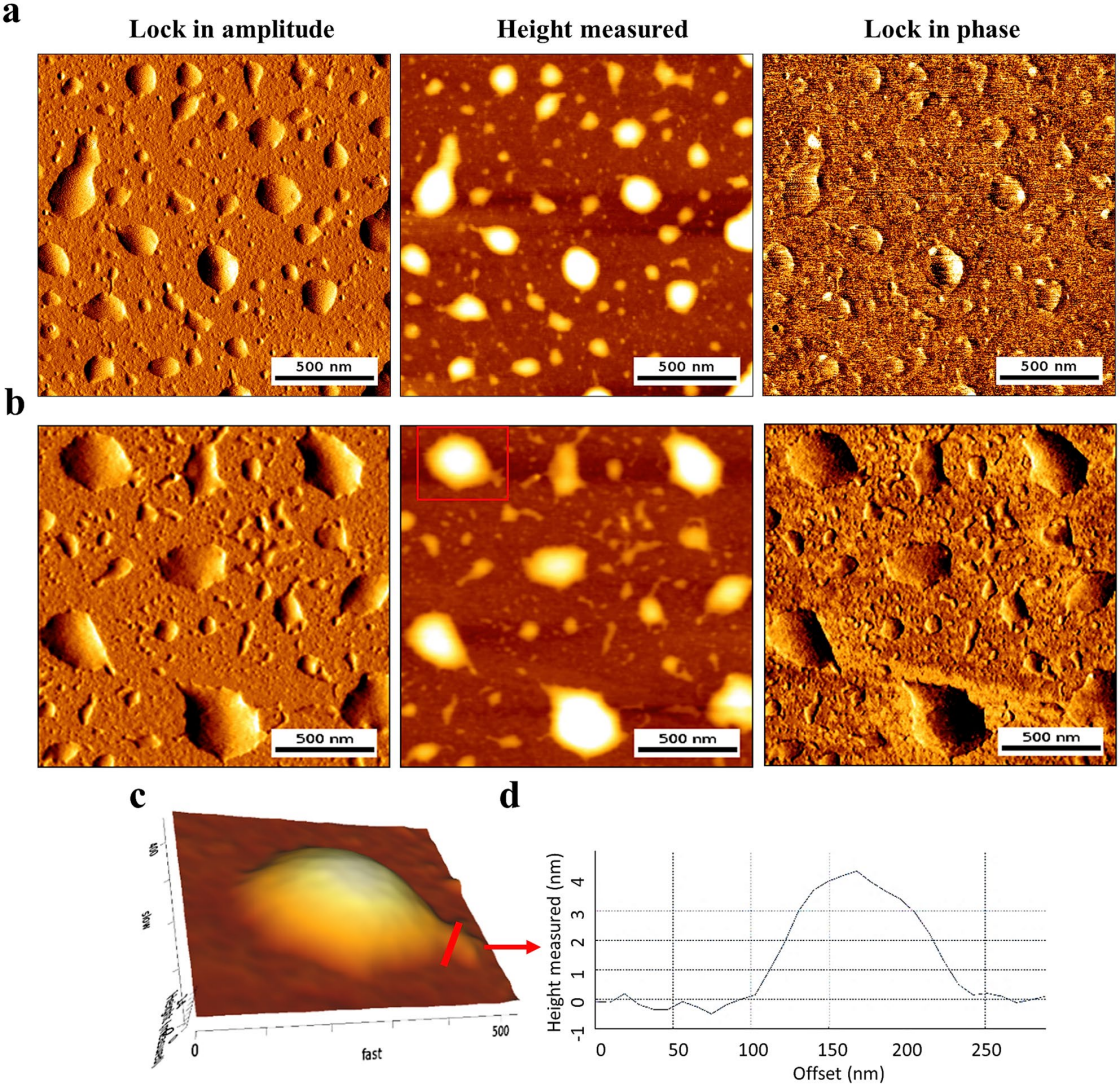
AFM evaluation of the complexes: lock-in amplitude, height measured and lock in phase images of (**a**) dendriplexes and (**b**) DPPC:CH-PAMAM lipodendriplexes (liposome to PAMAM dendrimer mass ratio 0.5/1; N/P ratio 12/1) at intermittent contact mode using NSC14 AlBS cantilever (scale bar represents 500 nm). (**c**) 3D height representative view of the single lipodendriplex (image size of 0.5 μm × 0.5 μm). (**d**) Cross-section view of spreading lipid layer (indicated by a red arrow) showing a height of 4 nm, representing a lipid bilayer.

Lipodendriplexes were prepared by incorporating DPPC:CH (85:15) liposomes with dendriplexes, as mentioned in the methods section. Atomic force microscopy was employed to evaluate the size and surface morphology of the complexes. Intermittent contact mode was used to taper the shear force on the particles and to avoid the damage by the cantilever needle. Results of the AFM images showed that dendriplexes and lipodendriplexes exhibited the sizes of 130.4 ± 10.7 nm and 259.6 ± 5.3 nm, respectively. The dendriplexes exhibited an almost round shape of the particles (Fig. 3a) while a slight broadening in shape, from spherical to oval, was observed in the case of lipodendriplexes particles. The slight change in shape was possibly due to a non-covalent interaction and adsorption of the lipid layer on dendriplexes and silica surface (Fig. 3b)^37^. Figure 3b also showed the spreading of the lipid layer along the particle surface, indicating the break and unfold of vesicles throughout the surface. The cross-section of spread layer showed a measured height of about 4 nm, confirming a lipid bilayer on the particle surface (Fig. 3c,d).

Surface roughness data also support the adsorption of negatively charged liposomes (*ζ-potential* *l*-7.7 ± 0.4 mV) on the highly positively charged surface of dendriplexes (*ζ-potential* + 25.6 ± 2.51 mV) to form lipodendriplexes system (*ζ-potential* + 7.5 ± 2.4 mV). The Rq values, considering 2.0 × 2.0 μm scan areas, indicated that the roughness of a lipodendriplexes was 1.71 nm lesser than the parent dendriplexes system (i.e., lipodendriplexes 3.4 ± 0.4 nm; dendriplexes 5.1 ± 0.9 nm), clearly depicting the adsorption, rupture and spreading of the lipid bilayers on the dendriplexes surface.

### pDNA transfection efficiency

**Figure 4:**
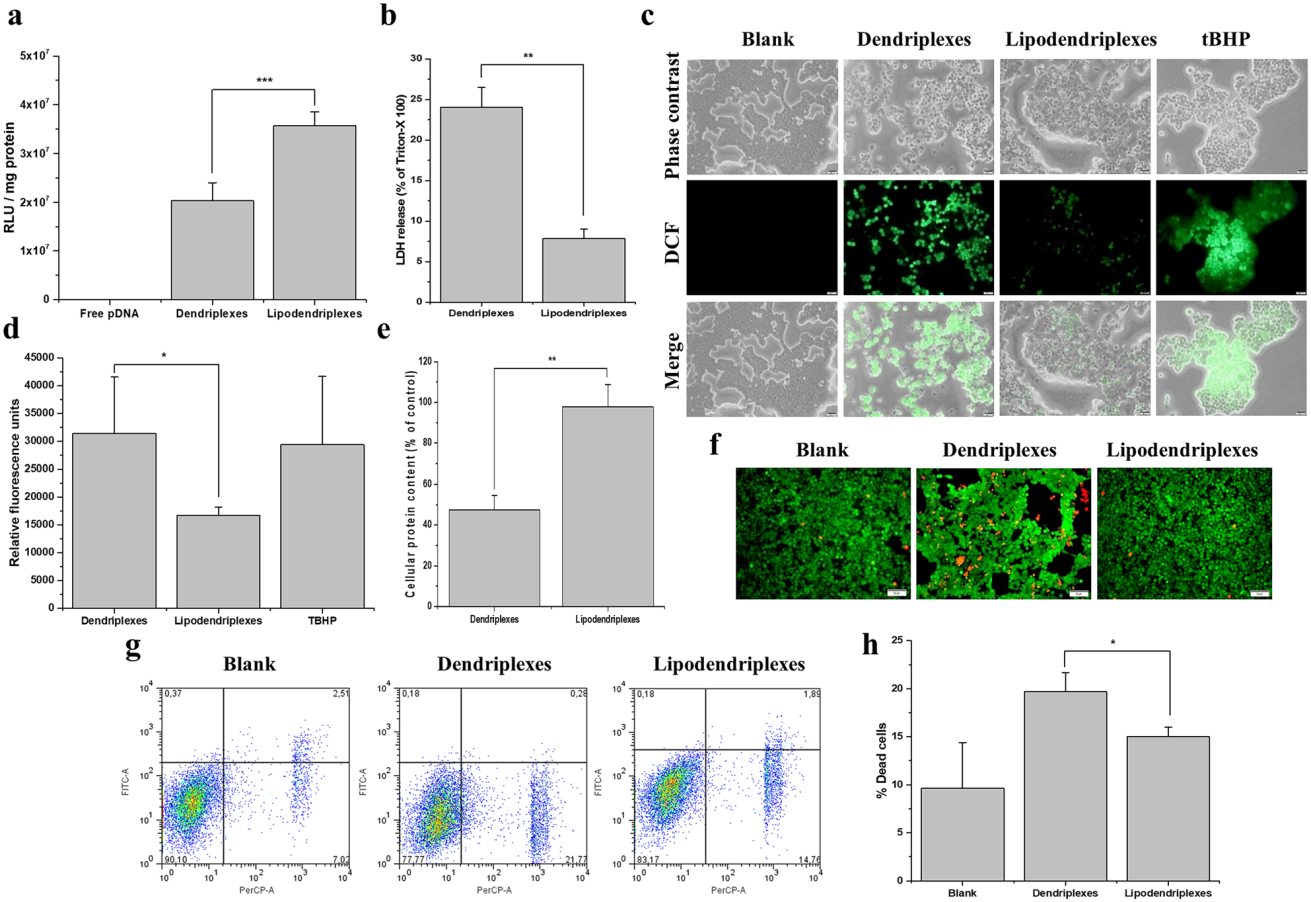
Transfection and toxicity analysis in HEK-293 cell line (**a**) pDNA transfection studies: lipodendriplexes exhibited significant improvement in transfection efficiency in comparison to dendriplexes and free pDNA. (**b**) LDH release assay: dendriplexes exhibited a significant increase in LDH leakage in comparison to lipodendriplexes. (**c**,**d**) Fluorescence micrograph and relative fluorescence units (RFU) of intracellular ROS generation after the addition of dendriplexes, lipodendriplexes and tBHP (scale bar represents 20 μm). Higher DCF (green fluorescence signals) was produced from H_2_DCFDA after the addition of dendriplexes to the cells in comparison to lipodendriplexes. (**e**) Cellular protein content determination (% of control) indicating a significant decrease in protein content of dendriplexes in comparison to lipodendriplexes. Untreated cells were used as control. (**f**) Live/dead assay of 2D cell culture: green signals of Calcein-AM indicating the live cells while the red channel of propidium iodide signals depicting the dead cells (scale bar represents 50 μm). (**g**) FACS micrographs indicating; left bottom = healthy cells, right bottom = dead cells, left top = early apoptotic cells, right top = late apoptotic cells. FITC-A and PerCP-A channels indicated the bandpass fluorescence filters for Annexin V/FITC (530/30 BP) and PI (695/40 BP), respectively. (**h**) Graphical representation of % dead cells after treatment with different complexes. Dendriplexes mediated cellular dead is more pronounced. Values are represented as mean ± SD (n = 3) and statistical significances are indicated as **p < 0.01, ***p < 0.001.

Transfection efficiency of complexes containing plasmid DNA encoding luciferase (pDNA) was conducted in HEK-293 cell line. Results of pDNA transfection efficiency demonstrated that lipodendriplexes exhibited significantly (p < 0.001) high transfection efficiency (3.5 × 10^7^ RLU/mg protein) as compared to its parent dendriplexes (1.8 × 10^7^ RLU/mg protein) (Fig. 4a). The cells treated with naked pDNA did not show any significant increase in transfection in comparison to untreated cells^38^.

### Lactate dehydrogenase release assay

It has been reported that the interaction of PAMAM dendrimers with anionic phospholipids of the cell membrane can induce the pore formation in the cell membrane and may result in the release of lactate dehydrogenase which can lead to cell death and apoptosis^39^. LDH release assay was performed to evaluate the cell membrane damaging effects of the complexes. The findings of LDH release depicted a significant (p < 0.01) damaging to the cell membrane by dendriplexes in comparison to lipodendriplexes treated cells (Fig. 4b).

### Reactive oxygen species generation assay

In the case of ROS assay, after the exposure of the complexes to the cells, the intracellular ROS generation in HEK-293 cells was evaluated. The dendriplexes, with exposed terminal amino groups, produced an increase in fluorescence signals of DCF as compared to that of the lipodendriplexes (Fig. 4c,d). This result portrayed that the positive charge surface of the PAMAM dendrimers played a critical role in ROS generation by the disruption of the mitochondrial electron transduction chain and enhanced O_2_ production^40^.

### Cellular protein content

To further evaluate the PAMAM related toxicity, cellular protein content was determined. From Fig. 4e it has been observed that the shielding of dendriplexes by lipids revealed a complete absence of cytotoxicity and exhibited 97% of cellular protein content (% of control). Contrarily, the cellular protein content of non-lipidic complexes were significantly (p < 0.01) less than that of lipodendriplexes system.

### Live/dead assay 2D cell culture

To visualize the cytotoxicity induced by different complexes, HEK-293 cells were stained by calcein-AM and PI mixture to observe the live and dead cells, respectively. Calcein-AM is a non-fluorescent dye and after permeation into the live cells, it is converted into a fluorescent compound by an intracellular esterase system to produce green fluorescence^41^. On the other hand, the dead cells can only be stained with PI because the nuclear membrane of the dead cells loss their integrity and allow the PI to bind the DNA to produce strong red fluorescence signals^42^. From Fig. 4f, it could be noted that the cells treated with lipodendriplexes exhibited higher green fluorescence signals and lower red fluorescence signals while the cells treated with dendriplexes showed intense red fluorescence signals in comparison to lipodendriplexes as well as the blank cells^43^.

### Annexin/PI flow cytometry

From the FACS graphs, it was observed that the incubation of complexes did not induce programmed cell death in HEK-293 cells, but we inferred that the presence of naked amino groups in dendriplexes may decrease the number of live cells by inhibiting cell proliferation (Fig. 4g). Figure 4h also depicted that the cells incubated with dendriplexes exhibited a significant increase in cell death (20%), compared to that of the lipodendriplexes treated and the blank cells. In apoptosis assay, the simplest way to evaluate cell death is the plasma membrane integrity. As mentioned in Sect. 6.6, a cell dies when it's plasma membrane becomes fragile allowing PI dye to enter the nuclear membrane to produce fluorescence. Therefore, the factors including the induction of nanoscale hole, transient leakage of cellular materials and significantly higher ROS generation by the primary amino groups of dendriplexes might led to cell death^40,44^.

### GFP transfection in spheroids

**Figure 5:**
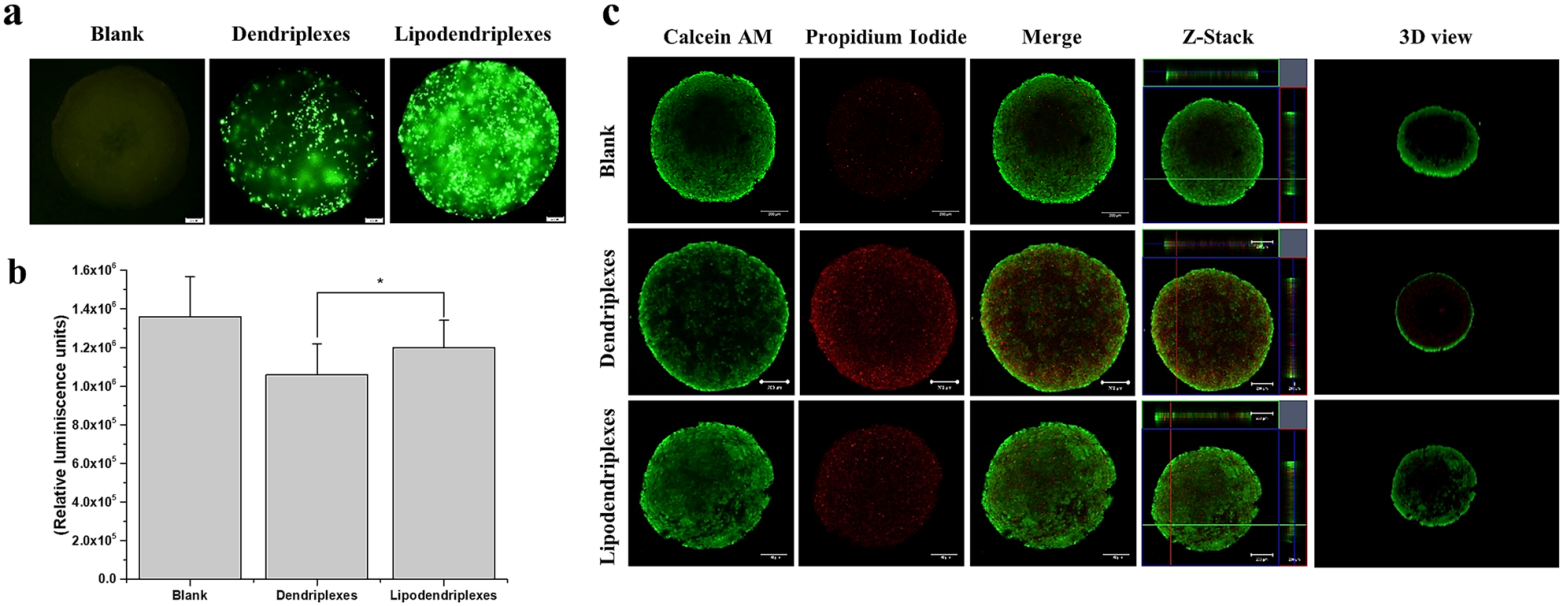
Transfection and toxicity analysis in HEK-293 3D spheroids (**a**) pCMV-GFP transfection studies in 3D spheroid: DPPC:CH-PAMAM lipodendriplexes (liposome to PAMAM dendrimer mass ratios 0.5/1; N/P ratio 12/1) exhibited higher transfection efficiency in comparison to dendriplexes. GFP expression is indicated by green fluorescence signals. Blank spheroids were without any treatment showing no fluorescence (scale bar represents 200 μm). (**b**) 3D cell viability assay: RLU signals are corresponding to the no. of live cells. Lipodendriplexes exhibited a significantly higher RLU values as compared to dendriplexes. (**c**) Live/dead assay of spheroids using CLSM: Calcein-AM green channel signals indicating the live cells while propidium iodide red channel signals depicting the dead cells (scale bar represents 100 μm). Values are represented as mean ± S.D (n = 3) and statistical significance is indicated as *p < 0.05.

The 3D spheroidal structure represents a similar morphological characteristic of in vivo tissues. The depletion of oxygen in the interior region of spheroids makes them insensitive for many therapeutic moieties. The interior region of spheroids having dormant cells and abundant extracellular matrix can also restrict the access of a nanocarrier system for required gene expression. From our experiment, it was observed that the lipid covering of dendriplexes enhanced the penetration and gene expression of the nanocarrier system in the spheroidal matrix system and demonstrated a superior GFP expression while a lower transfection efficiency was observed with the naked dendriplexes system (Fig. 5a)^45^. The results obtained with multicellular spheroid coincide with that observed in the 2D culture of HEK-293 cells. The transfection efficacy of the non-viral vector system depends on the cellular uptake and the efficient lysosomal escape of the nanocarrier system, but the lysosomal disruption by the naked dendriplexes system can induce lysosomal membrane permeabilization (LMP) leading to the cellular death and lower gene expression^39,46^.

### 3D cell viability assay

3D cell viability assay was performed to check the proliferation of the spheroids after the incubation of the complexes with HEK-293 spheroids. Results of Fig. 5b showed that lipodendriplexes exhibited high cell viability (1.2 × 10^6^ RLU) which was not significantly different from the spheroids having no treatment (1.3 × 10^6^ RLU), whereas significantly less cell proliferation was observed in the case of dendriplexes treated group (1.0 × 10^6^ RLU) indicating some arrest in cellular growth.

### Live/dead assay 3D culture

To further evaluate the cytotoxicity in 3D cell culture and to determine the live/dead cell ratio in the spheroids treated with different complexes, a live/dead viability assay was performed. Our findings showed that in comparison to dendriplexes, the spheroids incubated with lipodendriplexes exhibited more intense green fluorescence signals which was not significantly different from blank spheroids. On the other hand, higher PI signals were recorded in dendriplexes treated group suggesting the compatibility of lipodendriplexes with different cellular systems (Fig. 5c).

### Acute in vivo toxicity

#### Body weight and physio-social behaviour

**Figure 6:**
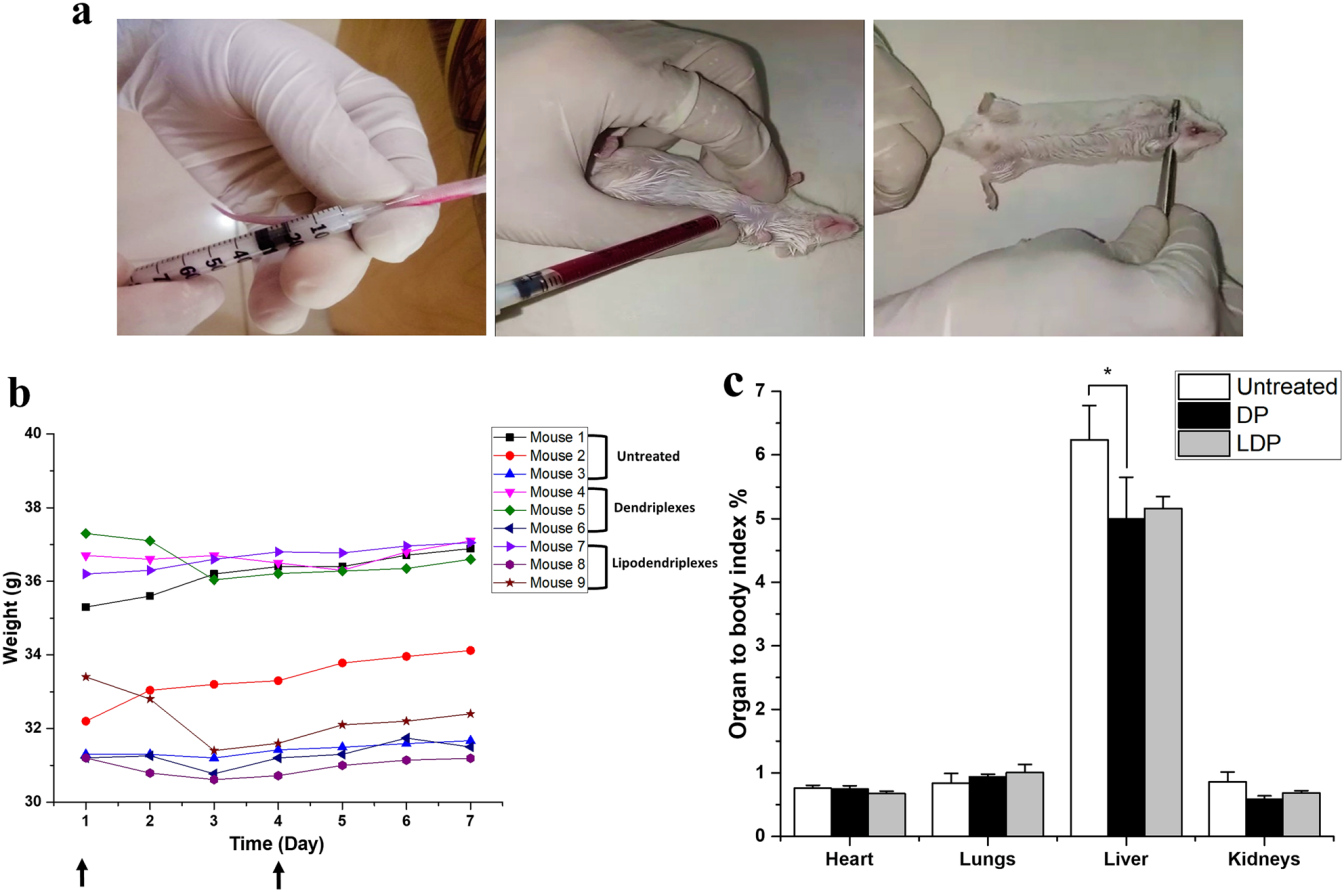
(**a**) Process of *i.v.* administration, blood collection by cardiac puncture and sacrifice of mice by cervical dislocation method, respectively. (**b**) Changes in body weight of mice for the 7 days in the untreated group (mice 1–3) and after the administration of the complexes containing 10 µg of pDNA (mice 4–6 dendriplexes and mice 7–9 lipodendriplexes of DPPC:CH-PAMAM; liposome to PAMAM dendrimer mass ratio 0.5/1 with N/P ratio 12/1). No changes in body weight of the mice were observed. (Arrows representing the repeated dose). (**c**) The organ to body index ratio of untreated group (white bar graph) and treated groups [dendriplexes (DP): black graph bar; lipodendriplexes (LDP): grey bar graph] after the sacrifice of animals by cervical dislocation. Values are represented as mean ± SD (n = 3) and statistical significance is indicated as *p < 0.05.

Acute in vivo toxicity was investigated in mice following *i.v.* injection of optimized complexes (dendriplexes and lipodendriplexes) containing 10 µg of pDNA by tail vein (Fig. 6a) and was compared to that of the untreated group. The animal body weight was monitored daily for 7 days. No significant difference in the body weight was found during the experimental period in both treated and untreated groups (Fig. 6b). The animal survival rate was 100% with no alteration in physical and social behaviours among the animals.

#### Organ to body index ratio

At the termination of the study, mice were sacrificed by cervical dislocation and the vital organs were removed to calculate the organ index of the heart, lungs, liver, spleen and kidneys. No significant change in the organ index was found after the treatment with dendriplexes and lipodendriplexes except for the liver to body index. The results of Fig. 6c showed that the liver to body index in the treated group (dendriplexes 4.9 ± 0.6% (p < 0.05); lipodendriplexes 5.1 ± 0.18%) was found to be less than the liver to body index of the untreated group (i.e. 6.2 ± 0.5%). PAMAM dendrimer-induced autophagy could be responsible for a reduced liver weight of the mice^47^.

#### Serum biochemical and hematological parameters

**Figure 7:**
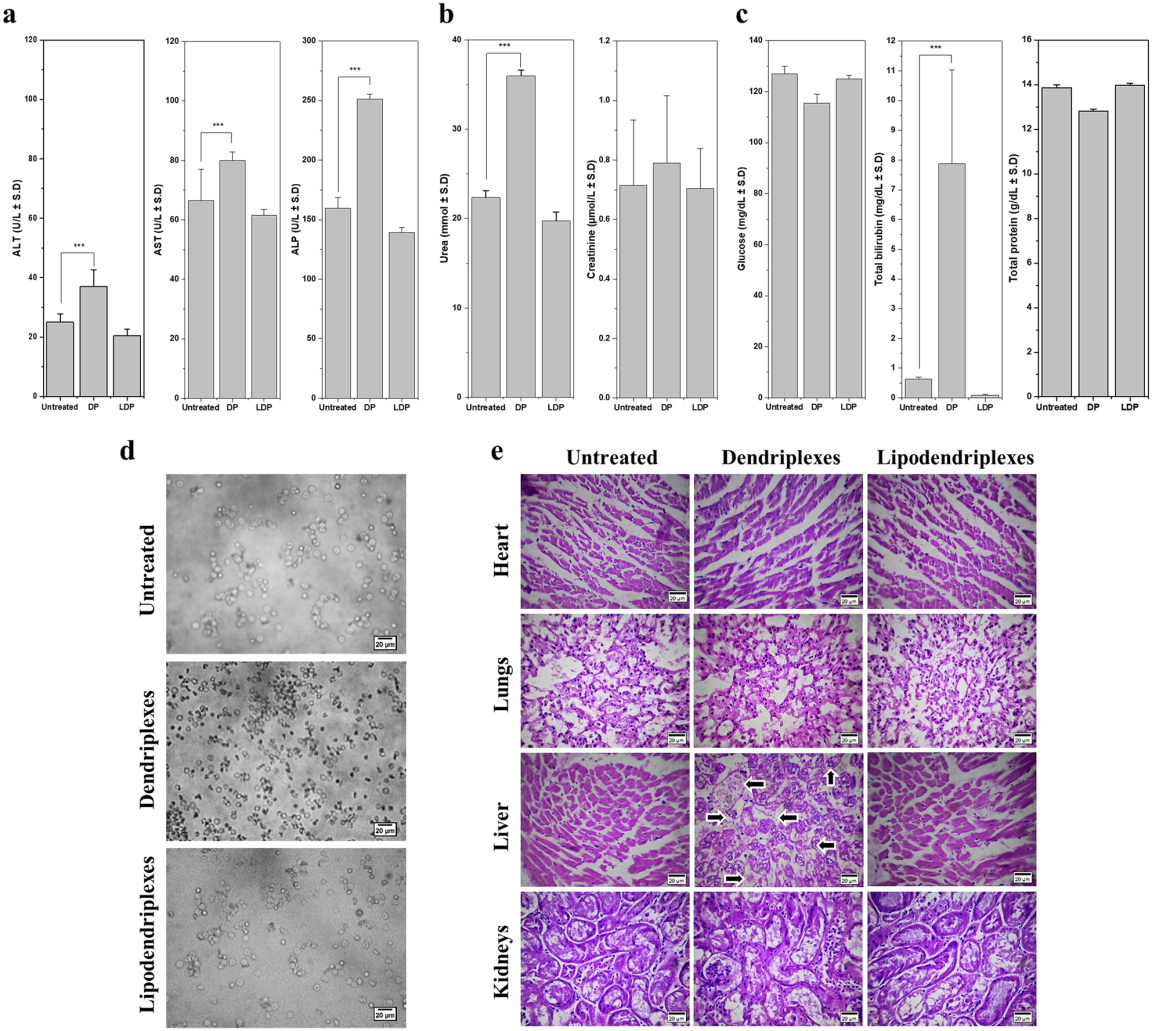
Typical serum biochemical markers, erythrocytes aggregation and histopathological investigations of an untreated group and after *i.v.* administration of the complexes containing 10 μg of pDNA (dendriplexes and lipodendriplexes of DPPC: CH-PAMAM; liposome to PAMAM dendrimer mass ratio 0.5/1 with N/P ratio 12/1). (**a**) Liver function tests (LFTs) parameters including ALT, AST and ALP levels. (**b**) Renal function tests (RFTs) parameters including blood urea nitrogen and creatinine levels. (**c**) Blood glucose, total bilirubin and total protein. (**d**) Ex vivo erythrocytes aggregation assay after treatment of complexes with 100 μl of erythrocytes suspension (2% v/v). Scale bar represents 20 μm. (**e**) H&E stained sections of vital organs from mice (heart, lungs, liver and kidney). All images were taken at ×40 magnification. Scale bar represents 20 μm. Values are represented as mean ± SD (n = 3) and statistical significances are indicated as **p < 0.01, ***p < 0.001.

Results of serum biochemistry markers showed a significant increase (p < 0.001) in the levels of alanine aminotransferase (ALT), aspartate aminotransferase (AST) and alkaline phosphatase (ALP) in dendriplexes-treated group, as compared to lipodendriplexes-treated and untreated groups as depicted in Fig. 7a. In the case of dendriplexes-treated group, hepatocellular injury and necrosis might be responsible for the increased leakage of ALT and AST in the bloodstream while an increase in the level of ALP suggested a bile obstruction in the bile duct. The level of blood urea nitrogen and creatinine was also higher in dendriplexes-treated groups, indicating the decreased efficiency of glomerulus or proximal tubule (Fig. 7b). These findings were in agreement with the studies conducted by Tang et al. and Wang et al.^48,49^. Significant elevation in total bilirubin (p < 0.001) was also associated with hepatotoxicity induced by dendriplexes in comparison to the other groups^50^, while some changes in blood glucose and total protein level by dendriplexes group were indicated their interference with the liver functioning and glucose metabolism as well (Fig. 7c)^51^.

In the hematological analysis, the major biomarkers including RBCs, HCT, WBCs, Hb, MCV, neutrophils, eosinophils, lymphocytes, monocytes, PLT, MCH, MCHC, MPV and percentage of neutrophils, monocytes, lymphocytes, eosinophils were monitored (Table 1). In the present study, the cationic dendriplexes triggered hemolysis of RBCs, as depicted by a significantly lower RBC (p < 0.05) and hematocrit count (p < 0.05) with a decreased level of Hb. The lipodendriplexes exhibited a slight decrease in Hb level in comparison to the untreated groups. Total leukocytes count was significantly increased in the both treated groups in comparison to the untreated group, which suggested the induction of some acute inflammatory response by the immune system. Neutropenia was observed in both treated groups while an increase in lymphocytes was monitored in dendriplexes treated group. In the case of dendriplexes-treated group, the PLT count was observed to be significantly (p < 0.01) higher stating the role of cationic PAMAM dendrimers in the activation of platelets and blood clot formation. This finding was also in agreement with the results obtained by Jones et al.^52^.

**Table 1:** Haematological parameters of mice in untreated and treated (dendriplexes, lipodendriplexes) group.

Blood parameter	Untreated	Dendriplexes	Lipodendriplexes
Hb (g/dl)	12.9 ± 1.4	12.1 ± 2.0	12.6 ± 1.6
WBCs (10^9^/l)	3.2 ± 1.2	4.4 ± 1.6*	6.4 ± 0.8**
RBCs (10^12^/l)	7.1 ± 1.2	6.6 ± 1.2*	7.19 ± 1.9
HCT (PCV) %	35.6 ± 6.8	33.5 ± 6.2*	39.2 ± 7.1*
MCV (fl)	49.6 ± 5.2	50.0 ± 6.0	54.5 ± 5.6
MCH (pg)	18.0 ± 2.7	18.0 ± 3.1	17.5 ± 1.8
MCHC %	36.3 ± 8.5	36.1 ± 7.4	32.1 ± 8.1
PLT (10^9^/L)	747.0 ± 25.2	794.0 ± 36.4**	731.0 ± 19.2
Neutrophils %	10 .0 ± 1.3	2.0 ± 1.4***	2.0 ± 1.0***
Lymphocytes %	80.0 ± 9.4	95.0 ± 10.7*	88.0 ± 8.1
Monocytes %	7.0 ± 1.6	2.0 ± 0.5***	8.0 ± 1.1
Eosinophils %	3.0 ± 0.8	1.0 ± 0.4	2.0 ± 0.4

Values are represented as mean ± S.D (n = 3) and statistical significances are indicated as *p < 0.05, **p < 0.01 and ***p < 0.001.

#### Erythrocytes aggregation assay

An erythrocyte aggregation analysis was performed to check the ex vivo behaviour of complexes on the blood cells. The dendriplexes treatment with erythrocytes exhibited a high level of aggregation with some hemolysis of the cells while the lipodendriplexes exhibited very low levels of interaction among the erythrocytes (Fig. 7d). Similar findings have been reported by Ewe et al.^53^.

#### Histopathology of vital organs

Histopathological analysis of major organs was done using H & E stained tissue sections (Fig. 7e). No discriminable changes were seen among the vital organs (heart, lung, and kidneys) of all groups. However, some scattered hepatocytic necrosis dots with pronounced vacuolization were observed in the dendriplexes treated group, indicating PAMAM dendrimers induced damages of the liver tissue. In the case of dendriplexes treated group, slight swelling in the renal glomerulus was also observed. This hepatic damage and glomerulus swelling induced by dendriplexes complied with the results; illustrated in Fig. 7b,c, showing the high bilirubin, blood urea nitrogen and creatinine levels. No renal toxicity was observed in the untreated and lipodendriplexes treated groups^54^.

Results of in vitro and acute in vivo toxicity demonstrated that the coating of cationic dendriplexes with liposomes markedly reduces the toxic properties of naked complexes such as their cytotoxicity towards cultured cells, blood cells and in vital organs as well.

### In vivo biodistribution analysis

#### Transgene expression and ex vivo imaging of vital organs

**Figure 8:**
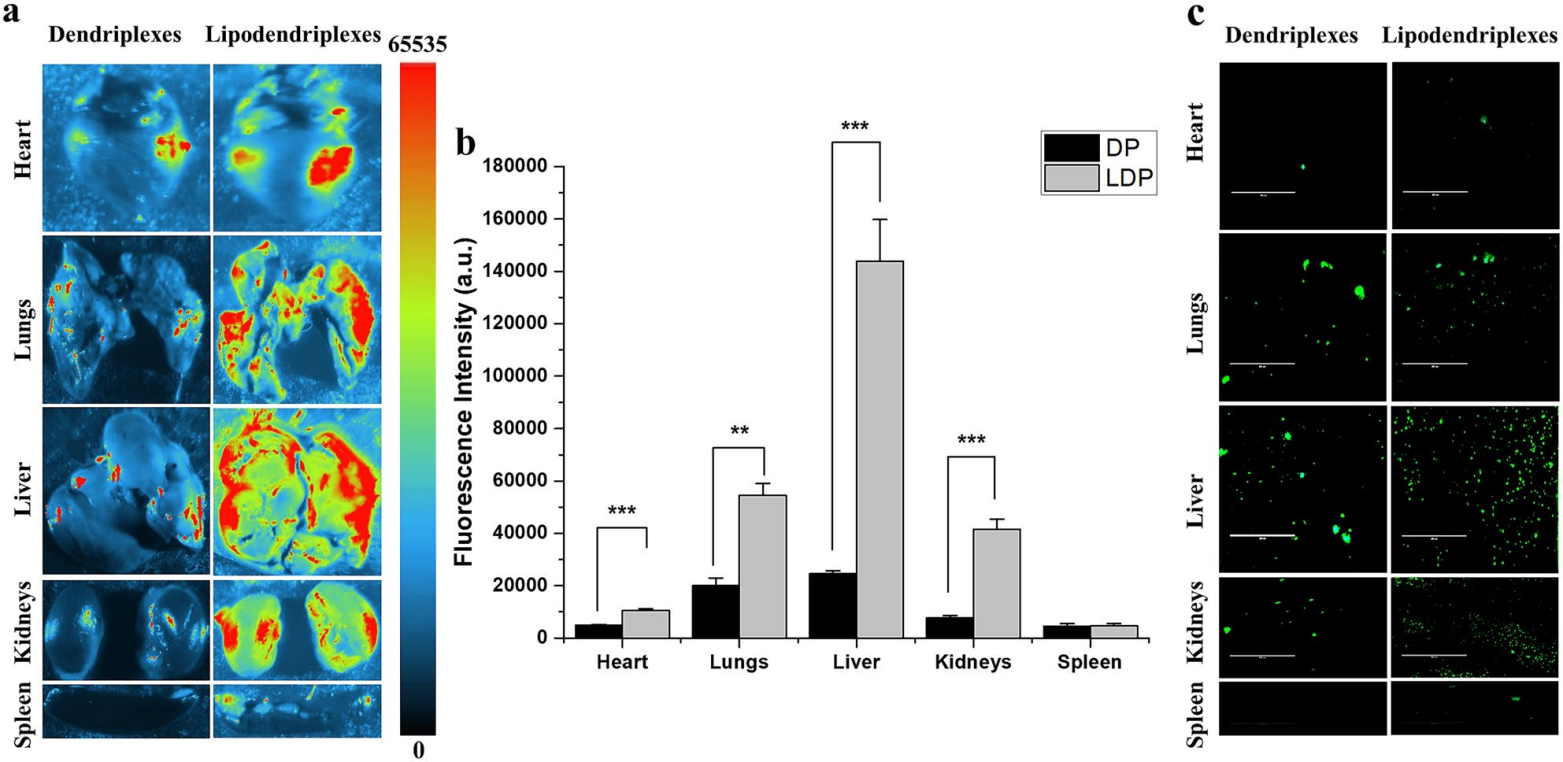
Ex vivo fluorescence analysis of vital organs (heart, lungs, liver, kidneys and spleen) after *i.v.* administration of the complexes containing 10 μg of pCMV-GFP (dendriplexes and lipodendriplexes of DPPC:CH-PAMAM; liposome to PAMAM dendrimer mass ratio 0.5/1 with N/P ratio 12/1). The mice were sacrificed 24 h after the administration of the complexes. The organs were collected carefully and washed with normal saline to remove any blood traces. (**a**) Ex vivo fluorescence images using UVP iBox Explorer2 small animal imaging system using wavelength filters for green fluorescence (ex.455 nm–495 nm, em.503 nm–523 nm). Fluorescence intensity is demonstrated by a color scale bar (red depicting maximum fluorescence intensity; 65535, while dark blue is minimum fluorescence intensity; 0) and (**b**) quantitative biodistribution (fluorescence intensity a.u) of pCMV-GFP labeled DNA complexes in vital organs. [Black graph bar represents dendriplexes (DP) and grey bar graph represents lipodendriplexes (LDP)]. Values are represented as mean ± SD (n = 3) and statistical significances are indicated as **p < 0.01, ***p < 0.001. (**c**) Ex vivo fluorescence imaging in the frozen thin section of dissected vital organs (heart, lungs, liver, kidneys and spleen). Green spots indicating the GFP expression in the cells (scale bar represents 100 μm).

The precise biodistribution of GFP labeled DNA complexes following *i.v.* administration was assessed in vital organs, using a fluorescence iBox Explorer2 imaging system. The images of the dissected organs were taken 24 h after the administration of the complexes to detect the fluorescence signals of GFP expression. The higher fluorescence signals of GFP expression were observed in liver, lungs, kidneys and heart as shown in Fig. 8a. In the case of dendriplexes the highest signals were detected in liver (24,606 ± 1047 a.u.) followed by the lungs (19,953 ± 3028 a.u. per organ), kidneys (7652 ± 1016 a.u.), heart (4923 ± 287 a.u.) and then in the spleen (4421 ± 932 a.u.). The liposome modification with dendriplexes significantly increased the fluorescence intensity in all organs, except the spleen, in comparison to naked dendriplexes. The fluorescence signals in the liver appeared to be highest with lipodendriplexes treatment (1,43,916 ± 15,876 a.u.), followed by lungs (54,517 ± 4552 a.u.), kidneys (41,582 ± 3804 a.u.), heart (10,483 ± 698 a.u.) and then in the spleen (4623 ± 932 a.u.) (Fig. 8b).

In vivo biodistribution of dendriplexes exhibited a low gene expression, which was due to the rapid plasma clearance and accumulation by the sites of the reticuloendothelial system (RES). The instability of dendriplexes was also due to the possible interaction of some anionic blood and cell membranes components with the terminal amino groups on the complexes, to get clearance from blood circulation in a few minutes^55^. The short blood circulation time limits the effective delivery of dendriplexes to different organs. However, the protective shielding of liposome over the dendriplexes stabilized the complexes against serum nucleases and exhibited a slower and efficient uptake into RES organs to provide long circulation time^56^. Therefore, a prolonged blood circulation by lipodendriplexes system maintains the efficient endosomal escape and transgene expression.

#### Frozen tissues GFP distribution analysis

To visualize the gene expression more clearly, the ex vivo fluorescence imaging was taken using the frozen thin sections of the major organs. A marked uniform distribution of GFP fluorescence was observed in the liver (hepatocytes and Kupffer cells), lungs and kidneys (Fig. 8c). The higher number of green fluorescent spots was observed in the lipodendriplexes treated group, which was in accordance with the result obtained from ex vivo imaging.

## Conclusion

In this study, we have used an efficient and safe gene delivery system by incorporating PAMAM based dendriplexes system with an optimized liposomal formulation of DPPC:CH (85:15). The strategy was to explore the full potential of the lipodendriplexes in vitro and in vivo environment. Therefore, after establishing the significant improvement in gene transfection and toxicity profile in vitro conditions, the complexes were investigated in vivo for biodistribution and acute toxicity assessment. Our results revealed that the incorporation of liposome with naked dendriplexes has essentially increased the cellular uptake of the complexes, which was confirmed by ex vivo fluorescence imaging of the dissected organs. Shielding of terminal amino groups of dendriplexes also improved the biocompatibility and eliminate the inherent dendrimer induced toxicity in vivo environment. From the findings, it could be concluded that the development of such a non-viral nanocarrier system could be considered for an efficient gene transfection with a better safety profile, both in vitro and in vivo delivery systems. However, further in vivo studies are required to get the full benefit of this system using different preclinical models for specific ligand-based delivery against different types of cancer and genetic disorders.
