# Correction: Lipodendriplexes mediated enhanced gene delivery: a cellular to pre-clinical investigation

**DOI:** 10.1038/s41598-025-32325-y

**Published:** 2026-01-08

**Authors:** Imran Tariq, Muhammad Yasir Ali, Muhammad Farhan Sohail, Muhammad Umair Amin, Sajid Ali, Nadeem Irfan Bukhari, Abida Raza, Shashank Reddy Pinnapireddy, Jens Schäfer, Udo Bakowsky

**Affiliations:** 1 Department of Pharmaceutics and Biopharmaceutics, University of Marburg, Robert‑Koch‑Str. 4, 35037 Marburg, Germany; 2 Punjab University College of Pharmacy, University of the Punjab, Allama Iqbal Campus, Lahore, 54000 Pakistan; 3 Department of Pharmaceutics, Faculty of Pharmaceutical Sciences, GC University Faisalabad, Faisalabad, Pakistan; 4 Riphah Institute of Pharmaceutical Sciences, Riphah International University, Lahore Campus, Lahore, Pakistan; 5 National Institute of Lasers and Optronics College, PIEAS, Islamabad, Pakistan; 6 CSL Behring GmbH, Emil‑von‑Behring‑Str. 76, 35041 Marburg, Germany; 7 Department of Pharmacy, Faculty of Health and Medical Sciences, University of Copenhagen, Copenhagen, Denmark

Correction to: *Scientific Reports* 10.1038/s41598-020-78123-6, published online 08 December 2020

This Article contains errors in Figure 7, where panel E incorrectly shows muscle tissue, instead of liver tissue for ‘Liver’, ‘Untreated’ and ‘Lipodendriplexes’.

The correct Figure 7 and accompanying legend appear below.

**Fig. 7:**
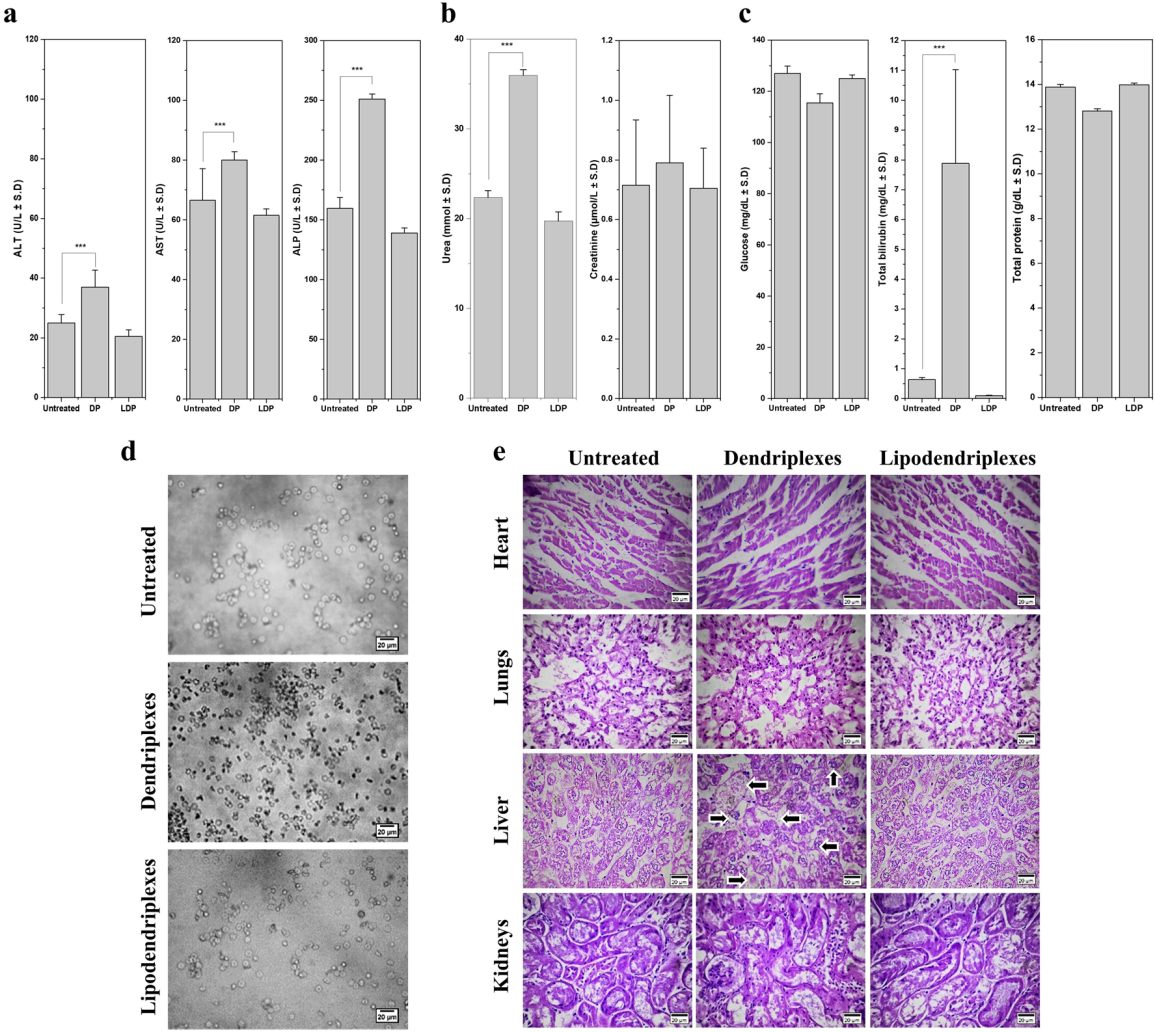
Typical serum biochemical markers, erythrocytes aggregation and histopathological investigations of an untreated group and after *i.v.* administration of the complexes containing 10 μg of pDNA (dendriplexes and lipodendriplexes of DPPC: CH-PAMAM; liposome to PAMAM dendrimer mass ratio 0.5/1 with N/P ratio 12/1). (**a**) Liver function tests (LFTs) parameters including ALT, AST and ALP levels. (**b**) Renal function tests (RFTs) parameters including blood urea nitrogen and creatinine levels. (**c**) Blood glucose, total bilirubin and total protein. (**d**) Ex vivo erythrocytes aggregation assay after treatment of complexes with 100 μl of erythrocytes suspension (2% v/v). Scale bar represents 20 μm. (**e**) H&E stained sections of vital organs from mice (heart, lungs, liver and kidney). All images were taken at × 40 magnification. Scale bar represents 20 μm. Values are represented as mean ± SD (n = 3) and statistical significances are indicated as **p < 0.01, ***p < 0.001.

